# A comparison of high-resolution and standard cardiovascular magnetic resonance myocardial perfusion imaging for the detection of myocardial ischaemia

**DOI:** 10.1186/1532-429X-13-S1-P82

**Published:** 2011-02-02

**Authors:** Neil Maredia, John P Greenwood, Catherine Dickinson, Aleksandra Radjenovic, Stephen G Ball, Sven Plein

**Affiliations:** 1 University of Leeds, Leeds, UK; 2 Leeds General Infirmary, Leeds, UK

## Objective

To compare high spatial resolution (*k-t* BLAST accelerated) with standard-resolution (SENSE-accelerated) myocardial perfusion CMR imaging for the detection of myocardial ischaemia.

## Background

First pass myocardial perfusion imaging by CMR may permit the detection of myocardial ischaemia with greater accuracy than single photon emission computed tomography (SPECT). Technical developments have led to improvements in myocardial perfusion CMR, particularly through accelerated data acquisition methods. These permit the acquisition of data at higher spatial resolution than conventional CMR techniques, though it is unknown whether this improves diagnostic performance.

## Method

Forty-seven patients with chest pain of potentially cardiac origin underwent two adenosine stress and rest myocardial perfusion studies on separate days. For acquisition of high-resolution data, *k-t* BLAST with eightfold acceleration and 11 training profiles was used, yielding a mean in-plane spatial resolution of 2.0 x 2.0mm and 103ms shot duration (1). The standard pulse sequence used twofold SENSE acceleration, yielding a mean 2.7 x 2.7mm in-plane resolution and 136ms shot duration. Otherwise the 2 pulse sequences were similar (T1-weighted saturation recovery, three short axis slices acquired). Each patient also underwent conventional SPECT and X-ray coronary angiography studies (2). Investigations were reported by observers blinded to the other test results.

## Results

**Figure 1:**
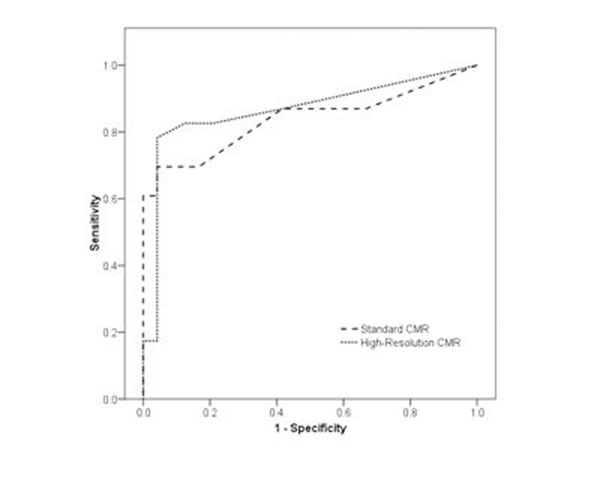
ROC Curve.

Dark rim artefact diameter was lower using high-resolution than standard CMR imaging (1.9 vs. 3.2mm, p<0.001) but image quality did not differ (median score 3 for both, p=0.8). The sensitivity and specificity of high-resolution CMR were 83% and 91%, standard CMR 74% and 88%, and SPECT 67% and 87% but no statistically significant differences were seen. Receiver operator characteristic curve analysis of summed stress perfusion scores revealed similar diagnostic accuracies for high-resolution and standard CMR techniques (areas under curve 0.87 and 0.84 respectively, p=0.58; figure 1). Respiratory artefacts affected 51% of high-resolution CMR studies; half of these occurred during the first pass of gadolinium. The presence of respiratory artefact did not have a significant impact on diagnostic accuracy.

**Figure 2:**
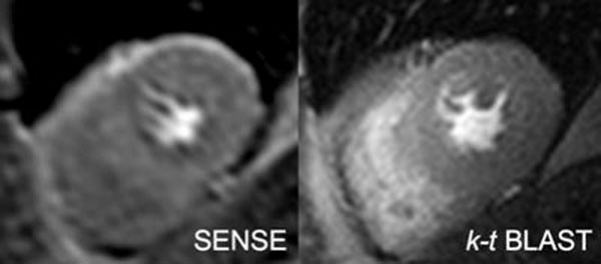
Sample images.

## Conclusion

High-resolution and standard CMR myocardial perfusion imaging show similarly high levels of sensitivity and specificity for the detection of X-ray angiography defined coronary artery disease. Both modalities are at least equivalent to SPECT imaging. Larger studies are needed to definitively determine whether diagnostic accuracy is improved with the high-resolution technique.

**Table 1:** Results.

	Sensitivity % (95% C.I.)	Specificity % (95% C.I.)	Positive Predictive Value % (95% C.I.)	Negative Predictive Value % (95% C.I.)
SPECT	67 (43-85)	87 (65-97)	82 (56-95)	74 (53-88)
Standard SENSE CMR	74 (51-89)	88 (67-97)	85 (61-96)	78 (57-91)
High-Resolution *k-t* BLAST CMR	83 (60-94)	91 (70-98)	91 (68-98)	84 (63-95)
